# Gemin5: A Multitasking RNA-Binding Protein Involved in Translation Control

**DOI:** 10.3390/biom5020528

**Published:** 2015-04-17

**Authors:** David Piñeiro, Javier Fernandez-Chamorro, Rosario Francisco-Velilla, Encarna Martinez-Salas

**Affiliations:** 1 Medical Research Council Toxicology Unit, Lancaster Rd, Leicester LE1 9HN, UK; 2 Centro de Biología Molecular Severo Ochoa, Consejo Superior de Investigaciones Científicas–Universidad Autónoma de Madrid, Nicolas Cabrera 1, Madrid 28049, Spain; E-Mails: j.fernandez@csic.es (J.F.-C.); rfrancisco@cbm.csic.es (R.F.-V.); emartinez@cbm.csic.es (E.M.-S.)

**Keywords:** Gemin5, RNA-binding proteins, translation control, IRES elements, snRNPs, picornavirus infection, Gemin5 proteolysis

## Abstract

Gemin5 is a RNA-binding protein (RBP) that was first identified as a peripheral component of the survival of motor neurons (SMN) complex. This predominantly cytoplasmic protein recognises the small nuclear RNAs (snRNAs) through its WD repeat domains, allowing assembly of the SMN complex into small nuclear ribonucleoproteins (snRNPs). Additionally, the amino-terminal end of the protein has been reported to possess cap-binding capacity and to interact with the eukaryotic initiation factor 4E (eIF4E). Gemin5 was also shown to downregulate translation, to be a substrate of the picornavirus L protease and to interact with viral internal ribosome entry site (IRES) elements via a bipartite non-canonical RNA-binding site located at its carboxy-terminal end. These features link Gemin5 with translation control events. Thus, beyond its role in snRNPs biogenesis, Gemin5 appears to be a multitasking protein cooperating in various RNA-guided processes. In this review, we will summarise current knowledge of Gemin5 functions. We will discuss the involvement of the protein on translation control and propose a model to explain how the proteolysis fragments of this RBP in picornavirus-infected cells could modulate protein synthesis.

## 1. Introduction

Ribonucleic acid-binding proteins (RBPs) are important players of gene expression control in all organisms, from Bacteria and Archaea to Eukarya. RBPs associate with nascent transcripts and are subsequently involved in all aspects of the RNA life cycle. Indeed, these factors co-ordinately regulate all steps of gene expression, from transcription, to RNA processing, export, localisation, translation and stability [1,2].

RBPs are defined through their ability to interact with their RNA target, which may be sequence specific or structure-dependent and occasionally sequence promiscuous. Often, RBPs interact with RNA, forming large ribonucleoprotein particles (RNPs). The composition of the RNPs undergoes dynamic remodelling during the cellular response to intra- and extra-cellular environment changes, leading to a reprogramming of their functional properties [3,4].

The number of novel RBPs is growing due to the implementation of highly sensitive identification methods. A large number of the proteins recently described as RBPs were previously shown to participate in cell metabolism pathways and other cellular events, unrelated to RNA life span. This is an indication of the multifunctionality of RBPs. Moreover, the diversity of activities performed by RBPs can also be indicative of their property to link processes occurring in the nuclear and the cytoplasmic compartment of the cell, as exemplified by RNA splicing factors, RNA transport and translation. In agreement with this, many RBPs, such as heterogeneous nuclear ribonucleoproteins (hnRNPs), are shuttling factors [5,6,7].

RBPs often are multidomain proteins containing motifs endowed with the capacity to interact with different targets, either proteins or nucleic acids (RNA and DNA). The interaction with the RNA partner takes place through the RNA-binding domain (RBD) of these proteins. The most common classes of canonical RBDs are the RNA recognition motif (RRM), the double-stranded RNA binding domain (dsRBD), the K homology domain (KH), the Pumilio homology domain (PUM-HD) and the zinc fingers [8]. Briefly, the RRM spans about 90 amino acids distributed in two conserved motifs, RNP1 and RNP2, organised in a four-strand antiparallel β-sheet backed by two α-helices. The dsRBD spans about 65–70 amino acids, folded into two α-helices packed against three-strand antiparallel β-sheets. The KH, which is about 70 amino acids, binds RNA through a cleft composed of two α-helices, a variable loop sequence, a conserved GXXG motif and a β-strand. The PUM-HD consists of eight PUF (Pumilio and FBF) repeats of a 36-amino acid motif. The entire domain forms a curved structure that interacts with RNA through the concave side. Finally, zinc fingers are a large and diverse class of domains with the common property of coordinating zinc. However, not all described RBPs harbour canonical RBDs, as noticed in the atlas of mammalian RBPs [9,10]. The presence of non-canonical RNA-binding sites (RBSs) appears to be a feature of many of the newly discovered RBPs.

**Figure 1:**
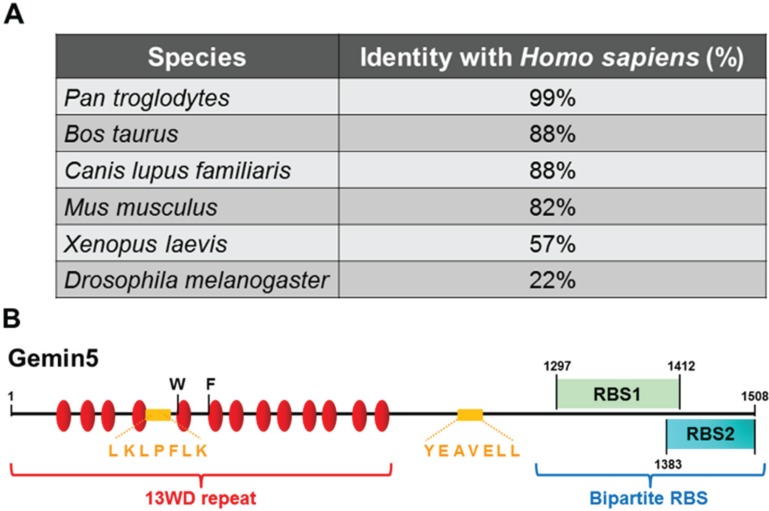
(**A**) Percentage of the identity of the human Gemin5 amino acid sequence with other species; (**B**) schematic representation of the functional domains of Gemin5. Red ovals depict WD repeat domains, green rectangles RBS1 and blue rectangle RBS2. The 4E-binding motifs are marked by orange letters and the position of W286 and F338 residues by black letters. Numbers indicate amino acid positions.

Proteins performing the same function in organisms belonging to different kingdoms are evolutionary conserved. This property applies to factors controlling basic aspects of cell proliferation, such as DNA replication, RNA transcription, protein synthesis and RNA or protein turnover. Among other examples, the minimal survival motor neuron (SMN) ribonucleoprotein assembly system, composed of SMN protein and Gemin2, is conserved in all eukaryotic organisms with the exception of *S. cerevisiae* [11]. The increased complexity of the SMN complex takes place later in evolution. In mammals, the SMN complex consists of the SMN protein, the Gemin proteins designated 2 to 8 and Unr-interacting protein (Unrip) [12,13,14]. The SMN complex is responsible for the assembly of the seven-member (Sm) core proteins arranged in a heptameric B-D1-D2-F-E-G-D3 ring surrounding the snRNAs to generate uridine-rich snRNPs [15], which are the essential components of the spliceosome [16,17]. Disruption of SMN complex function can cause motor neuron disease [18]. Gemin5, the RBP of the SMN complex, is lacking in *C. elegans*, but is retained in evolutionarily distant organisms, such as the soil amoeba *D. discoideum* and the green alga *O. tauri* [11,19]. In fact, alignment of the amino acid sequence of Gemin5 shows that the protein is highly conserved in mammals relative to the human sequence (82% identity in *Mus musculus*; up to 99% in *Pan troglodytes*). Sequence conservation drops to 57% in *Xenopus laevis*, while the ortholog of Gemin5 in *Drosophila melanogaster* only shares 22% identity with human (Figure 1A) [20,21].

## 2. Cellular Processes that Depend on Gemin5 Function

Gemin5 is an abundant protein, predominantly distributed in the cell cytoplasm, as shown by immunofluorescence microscopy and subcellular fractionation studies [22]. Co-localisation of Gemin5 with the SMN complex in nuclear gems, but not in Cajal bodies, has been detected within the nucleus [23]. In addition, the protein Gemin5 localises in stress granules within the cytoplasm in response to arsenite treatment and heat shock [24]. These differences in the cellular distribution of the protein are indicative of either a capacity to perform distinct functions or act as a vehicle of its target RNA shuttling to different cell compartments.

Gemin5 was described as a peripheral RBP of the SMN complex [22]. The protein recognises and delivers the small nuclear RNAs (snRNAs) to the SMN complex, allowing the assembly of the small nuclear ribonucleoproteins (snRNPs) [25]. In the fly fruit, Gemin5/Rigor mortis is one of the SMN complex proteins, together with Gemin2 and Gemin3. Subcellular distribution of the SMN complex proteins is colocalised in the cytosolic granule U bodies, containing uridine-rich small nuclear ribonucleoproteins (U snRNPs). In Drosophila germline cells, U bodies associate with P bodies. U snRNPs play a key role in pre-mRNA processing in the nucleus [26]. Gemin5/Rigor mortis protein has also an important function in development; its loss is lethal at the larva stage [20,21].

Beyond its role in the snRNPs assembly, Gemin5 has been shown to participate in the alternative splicing process and in tumour cell motility. The MDA-MB-435 tumour cell line modified to maintain the metastatic property (C-100) or to supress it (H1-177) was used to analyse the global mRNA splicing profile [27,28]. This study showed a differential splicing profile between cell lines, which was dependent on Gemin5. Overexpression of Gemin5 in C-100 recovered the splicing profile observed in H1-177 and decreased the motility of the cells. In contrast, reduction of Gemin5 levels in H1-177 cells by siRNA interference induced an increased motility of the cells.

More recently, it has been shown that Gemin5 can bind with two genetically distant viral internal ribosome entry site (IRES) elements and that this factor downregulates translation [29,30]. Finally, presumably unrelated to its RNA-interacting capacity, Gemin5 has been reported to be a scaffold protein, playing a role in the assembly process of the complex containing apoptosis signal-regulating kinase 1 (ASK1), stress-activated protein kinase 1 (SEK1) and c-Jun NH2-terminal kinase 1 (JNK1) proteins, which are involved in H_2_O_2_ and tumour necrosis factor-α (TNFα) driven apoptosis [31].

## 3. The Role of Gemin5 in the Biogenesis of snRNPs

The stepwise pathway leading to snRNP biogenesis takes place in the cytosol. Briefly, Gemin5 interacts with snRNA precursors (pre-snRNA), and the resulting complex is added to the SMN complex, which assembles the snRNP [32]. There are five snRNPs with different sequences, each of them derived from a single pre-snRNA [33]. Subsequently, snRNPs are transported from the cytosol to the nucleus, within which they participate in the splicing process.

Gemin5 can interact directly with the SMN protein and several Sm core proteins, although this interaction does not determine the stability of the SMN complex [12,34]. Gemin5 cooperates with the SMN complex to assemble the Sm core proteins during snRNPs biogenesis [22,25] in a transitory manner, possibly explaining why earlier studies failed to identify Gemin5 in the SMN interactome [35]. Decreased Gemin5 levels by RNA interference results in a reduction of both the capacity of the SMN complex to bind snRNAs and the ability to assemble Sm cores on snRNAs [25,34], indicating that Gemin5 plays a crucial role in the snRNPs biogenesis.

Gemin5 is a 170-kDa protein with distinct functional domains (Figure 1B). A 13WD repeat domain is placed at the amino terminal end, whereas a bipartite non-canonical RNA binding site (RBS1 and RBS2) is located at the carboxyl end of the protein [22,30]. The region of the protein involved in the interaction with snRNAs has been mapped to the 13 WD repeat domain. Specifically, the fifth WD repeat domain is responsible for the interaction, as shown by RNA-mediated radical probing and mass spectrometry [36]. Additionally, the structural integrity of this domain is crucial to establish the interaction. Mutational analysis has shown that the W286 residue is important for this interaction. In vertebrates, this residue is conserved within Gemin5 orthologs, although it is not generally conserved at this position in the other WD repeat domains. Currently, it is not known whether the residue W286 interacts directly with the pre-snRNA or if it is important to generate an appropriate structural platform for the binding with the snRNAs [36]. Nonetheless, this particular residue may be an adaptation for the specialised function of pre-snRNAs binding.

**Figure 2:**
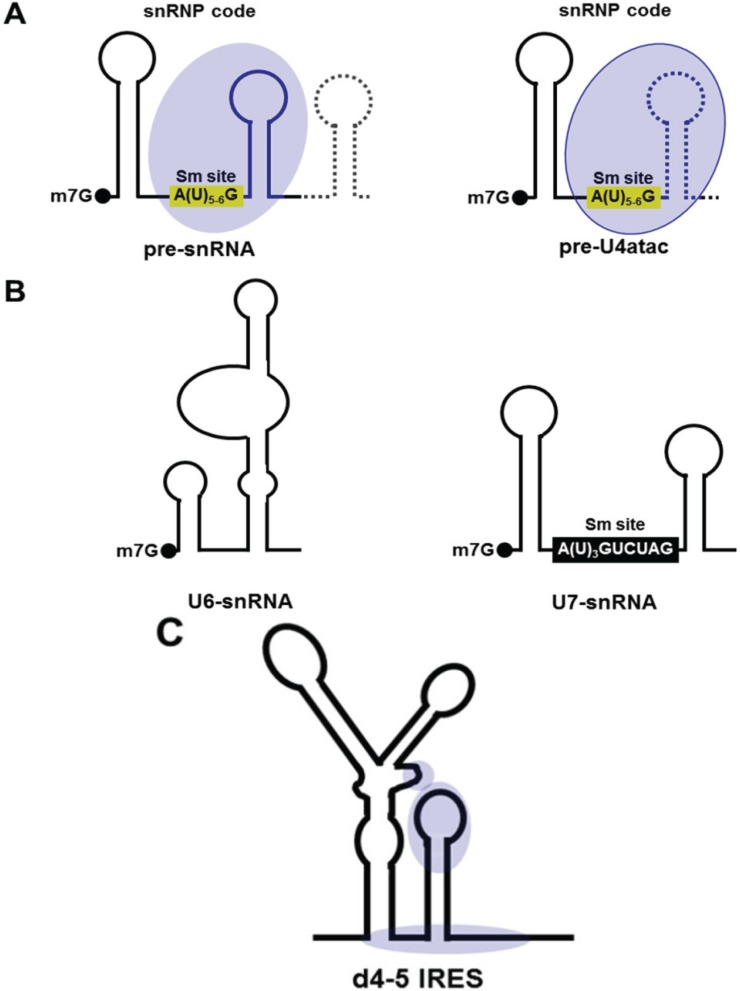
Schematic representation of the Gemin5 RNA targets. (**A**) The RNA target model represents U1, U2, U3, U11, U12 and U5 pre-snRNAs (left) and pre-U4atac snRNA (right). A yellow box depicts the canonical Sm site. The dotted line represents the RNA region that is removed in the mature form of snRNAs. A blue oval represents the Gemin5 binding site. (**B**) Schematic representation of U6 and U7-snRNAs, which are not targets of Gemin5. A black box represents the non-canonical Sm site. (**C**) Schematic representation of the binding sites of Gemin5 on domains 4 and 5 of the foot-and-mouth disease virus (FMDV) IRES (blue ovals).

The identification of the Gemin5-snRNA intermediate was shown after induction of cell stress by arsenite in Hela cells. In this case, Gemin5 binds snRNA independently of the SMN complex and without a requirement for ATP. In contrast, ATP hydrolysis is required in the process of snRNP assembly [24]. Gemin5 recognises a specific structural feature present in the pre-snRNA, designated the snRNP code, which includes the Sm site [A(U)_5-6_G] and at least one stem-loop (SL) located at the 3' end of the Sm site (Figure 2A). Pre-snRNAs U1, U2, U3, U4, U4-atac, U11, U12 and U15 contain this feature. All of these RNAs have been identified as interacting with Gemin5 by high throughput sequencing [32]. The mature form of snRNAs interacts with Gemin5 in RNA-binding assays, with the exception of U4-atac. This is presumably due to the lack of the 3' SL in the mature U4-atac snRNA. Other examples of snRNAs that do not interact with Gemin5 are U6 and U7 snRNAs (Figure 2B). U6 snRNA lacks the snRNP code [37]. U7 snRNA contains a non-canonical Sm site (AUUUGUCUAG), and it is involved in histone RNA processing, but not in snRNPs biogenesis [38]. Thus, the sequence of the Sm site and the presence of the 3' SL in snRNAs are crucial for their recognition by Gemin5.

In summary, Gemin5 is a RNA binding protein that uses its fifth WD repeat domain to interact with snRNAs in order to trigger the assembly of snRNPs by the SMN complex. Hence, the 13WD repeat domain of Gemin5 performs a defined function in snRNPs biogenesis.

## 4. Gemin5 Crosstalks with the Translation Machinery

Currently, several independent observations link Gemin5 with translation control events. First, Gemin5 has been described to interact with the eukaryotic initiation factor 4E (eIF4E) [39]. Furthermore, Gemin5 and eIF4E co-localise in cytoplasmic P-bodies in human osteosarcoma U2OS cells. It is interesting to note that the P-bodies are associated with transition from active translation to mRNA degradation [40]. Second, it has been reported that Gemin5 displays cap-binding capacity [41]. Third, Gemin5 interacts directly with specific domains of the foot-and-mouth disease virus (FMDV) and hepatitis C virus (HCV) IRES elements and acts as a downregulator factor of both cap-dependent and IRES-driven translation [29].

### 4.1. Gemin5 Implications on Cap-Dependent Translation Initiation

The vast majority of Gemin5 is outside of the SMN complex [24], suggesting that, beyond its role in the SMN complex, the protein may participate in other cellular events.

Quantitative proteomic analysis of the human cap-binding complex led to the identification of Gemin5 as a novel eIF4E-binding partner [39]. Gemin5 harbours a 4E-binding motif (YXXXXLΦ), present in a number of eIF4E-interacting factors, including eIF4GI, eIF4GII and eIF4E-BP proteins [42]. Gemin5 has two putative eIF4E-binding motifs, LKLPFLK and YEAVELL (Figure 1B). The first one is localised between the fourth and the fifth WD repeat domain (residues 265–271), while the second one occurs within residues 992–997. Mutational analysis of these motifs showed that a direct interaction of Gemin5 with eIF4E required the integrity of these motifs [39]. Since both eIF4E and Gemin5 are RBPs, it could not be discarded that RNA bridges participate in this complex.

As mentioned above, Gemin5 has cap-binding capacity. The cap-recognition motif resides in the 13WD repeat domain according to cap-affinity chromatography of C-terminal and N-terminal truncated proteins [41]. Since eIF4E is the protein that recognises the cap structure, it could be argued that the identification of Gemin5 in the cap-affinity chromatography is due to its eIF4E-binding capacity [39]. However, Gemin5 was crosslinked to radiolabeled cap-structure after UV irradiation [41], suggesting a direct cap-binding capacity independent of eIF4E. Currently, the three-dimensional structure of Gemin5 remains elusive. Notably, it has been suggested that the interaction with the cap requires a complete structural organisation of the 13WD repeat domain [41]. Moreover, modelling of Gemin5’s 13WD repeat domain using the protein homology/analogy recognition engine (PHYRE) algorithm combined with site-directed mutagenesis pointed at two aromatic amino acids, W286 and F338 (Figure 1B), located in the fifth and sixth WD repeats, as the putative cap-interacting residues. The relevance of these residues was confirmed by mutational analysis. Replacement of F338 by alanine reduces the binding, and substitution of W286 by alanine abolishes the cap-binding capacity of the protein [41]. Hence, these aromatic amino acids and a strict structural requirement of the 13WD repeat are key features to establish the Gemin5-cap interaction.

The ability of Gemin5 to interact with both eIF4E and the cap structure suggests that Gemin5 may interfere with the recruitment of the eIF4F complex to mRNAs. This hypothesis may explain the downregulatory effect of the protein in cap-dependent translation initiation. Further studies will be necessary to elucidate the mechanism underlying the role of Gemin5 in cap-dependent translation control.

### 4.2. Gemin5 Role on IRES-Dependent Translation Initiation

Two independent approaches, riboproteomic analysis and immunoprecipitation of photocrosslinked factors, showed that Gemin5 interacts with the IRES elements of two RNA viruses, FMDV and HCV [29]. The interaction of Gemin5 with the picornavirus FMDV IRES element was subsequently studied in further detail. Functional analysis involving the expression of Gemin5 in cell-free systems showed that increasing amounts of protein inhibited IRES-dependent expression in a bicistronic mRNA. Moreover, Gemin5 depletion in BHK-21 and HEK293 cell lines by either shRNA or siRNA showed a downregulatory role of the protein in IRES-dependent translation [29]. Interestingly, the IRES downregulatory effect was mapped to the most C-terminal region of the protein, containing the RBS2 domain (Figure 1B) [30]. The capacity of Gemin5 to out-compete polypyrimidine tract binding protein (PTB) from its binding site [43,44], a protein enhancing IRES-dependent translation initiation, could explain its function as a downregulatory factor of the IRES-dependent translation initiation [45].

The FMDV IRES consists of five structural RNA domains (1–2, 3, 4 and 5) [46]. Domain 2 contains a conserved pyrimidine tract that provides the PTB binding site [47]. Domain 3 is a self-folding cruciform structure that contains evolutionarily conserved motifs, essential for IRES activity [48,49,50]. Domain 4 provides the binding site for eIF4G [51,52]. Finally, domain 5 located at the 3' end of the IRES element, consists of a hairpin of nine base pairs, followed by a single-stranded region that contains a conserved polypyrimidine tract [53]. This relatively small domain 5 provides the binding-site for eIF4B and PTB [54,55] and other RBPs, including Gemin5 [56]. It is noteworthy that Gemin5 interacts specifically with the hairpin of domain 5 (Figure 2C) in immunoprecipitation assays of photocrosslinked complexes, but not with the single-stranded region of this domain. The interaction with the IRES element allows sequence flexibility [45], unlike what happens in the Gemin5-snRNA interaction [25]. Additionally, selective 2'-hydroxyl acylation analysed by primer extension (SHAPE) footprints using purified protein indicated that Gemin5 protects a few residues of domain 4, the spacer region between domains 4 and 5 and the hairpin of d5 (Figure 2C) [45]. Hence, the Gemin5-IRES interaction seems to have a specific structural requirement. The property to recognise double-stranded RNA is shared with other RBPs, such as human Staufen1 (Stau1) [57,58,59], the *Xenopus* homologue of human TAR-RNA binding protein (TRBP) (xlrbpa) [60], TRBP [61] or microprocessor complex subunit DGCR8 [62,63].

**Figure 3:**
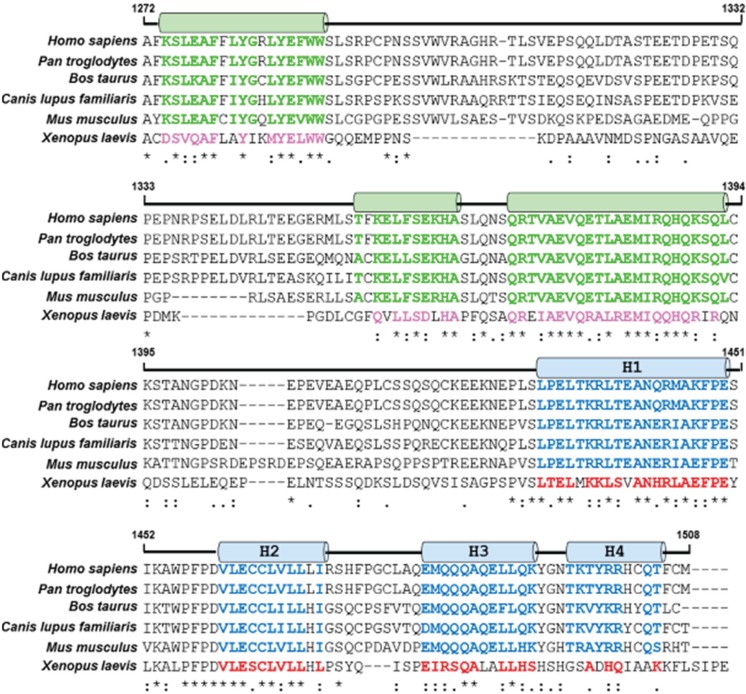
Multiple sequence alignment of Gemin5 sequences spanning the C-terminal region of the protein. Polypeptide RBS1 spans residues 1297 to 1412, while RBS2 spans residues 1383 to 1508. Green or blue cylinders depict the helical region of RBS1 or RBS2, respectively. The amino acids conserved in RBS1 or RBS2 in mammals are depicted by green or blue letters, respectively. Pink or red letters depict amino acids conserved in the *Xenopus* sequence in RBS1 or RBS2, respectively.

Dissection of the Gemin5 protein into various fragments has shown that the C-terminal region of Gemin5 has RNA binding capacity [30,45]. It is noteworthy that the C-terminal region of the protein is sufficient to bind with the IRES element. The region harbours a non-canonical bipartite RNA-binding site (RBS) (Figure 1B). Both sites, designated RBS1 and RBS2, have the capacity to interact with domain 5 of the IRES, although the RBS1 displays higher RNA-binding affinity than RBS2. Structural analysis of the RBS1 polypeptide, which spans residues 1297 to 1412, by NMR has shown that this region is largely unstructured [30], but it may contain three short helical regions (Figure 3). The presence of this unstructured region may confer flexibility to the Gemin5 protein. This characteristic may represent an advantage for Gemin5 by which the protein can select the best conformation among the ensemble of the protein structures to recognise different ligands.

Currently, there is no experimental evidence of the structure of the RBS2 region (residues 1383 to 1508). Prediction models for the C-terminal portion of the protein have shown that RBS2 comprises four helices (H1–4). Interestingly, the helix H2 is enriched in leucine, and the helix H3 is a glutamine-rich sequence [30] (Figure 3). Proteins with leucine-rich and, particularly, glutamine-rich motifs can mediate protein-RNA interactions, as reported for Leucine-rich PPR motif-containing protein (LRPRPC) [64] and TIA 1 cytotoxic granule-associated RNA binding protein [65]. Multiple sequence alignment of Gemin5 sequences from various organism spanning both RBS1 and RBS2 regions shows that helical regions present in the RBSs are conserved in mammals (Figure 3). Thus, it is tempting to suggest that these features may explain the capacity of Gemin5 to interact with the IRES elements. With the exception of the FMDV IRES, there is no information regarding the capacity of this protein to modulate internal initiation of translation driven by other IRES elements. It is expected that future studies aimed at addressing this question will expand the knowledge of the functions of this protein in gene expression control.

## 5. Processing of Gemin5 in Virus-Infected Cells

Picornaviruses induce a shutdown of cap-dependent translation in infected cells [66]. Cleavage of host factors, many of them RBPs, by viral-encoded proteases profoundly alters several processes critical for cell viability, including transcription, nucleo-cytoplasmic transport, RNA granules composition and global protein synthesis [67]. However, translation of picornavirus RNA is resistant to cap-dependent inhibition [68,69]. Picornavirus and other positive-strand RNA viruses subvert the host translational machinery to promote translation of the viral genome using a cap-independent mechanism [70,71]. These viruses highjack the translation machinery and evade translation inhibition, taking advantage of IRES elements. These RNAs recruit the 40S ribosomal subunit internally by a process guided jointly by RNA structural motifs, a subset of eIFs and a number of RBPs [72,73].

**Figure 4:**
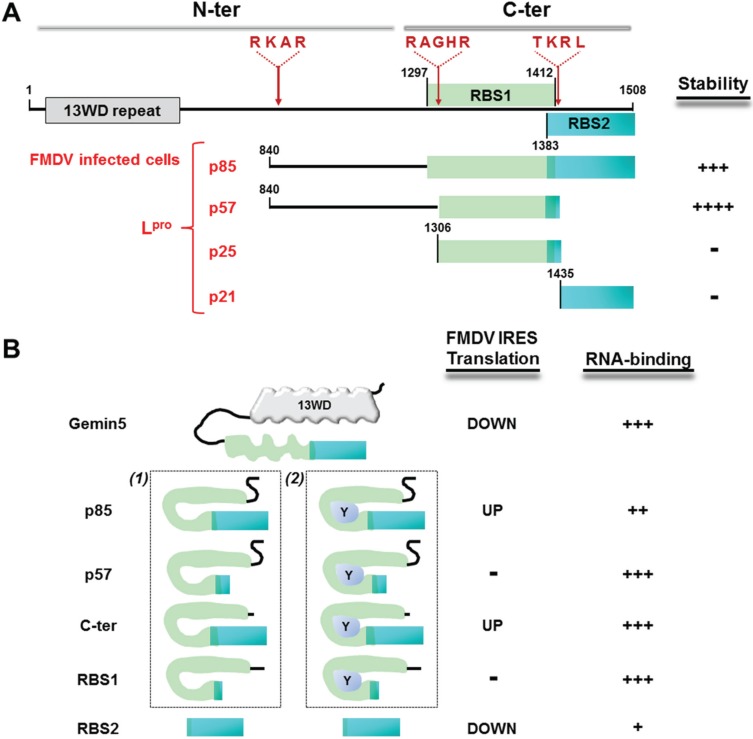
(**A**) Schematic representation of Gemin5 cleavage products observed during viral infection. N-ter and C-ter indicates amino-terminal and carboxi-terminal regions, respectively. The sequences recognised by L^pr^°, RKAR, RAGHR and TKRL are indicated. ++++ depicts the higher stability of p57, compared to p85; +++ indicates undetected in infected cells. (**B**) Hypothetical model of the role of Gemin5 in IRES-dependent translation. (1) and (2) represent two alternative mechanisms possibly occurring in infected cells. Y (blue oval) indicates an unknown cellular factor possibly interacting with the proteolytic products. The capacity to regulate translation is indicated by on/off and the intensity of the IRES interaction by +, ++ or +++.

Given that one of the factors proteolysed in picornavirus-infected cells was the RNA helicase of the SMN complex, Gemin3 [74,75], the stability of Gemin5, another RBP component of this complex, was analysed in cells infected with several picornaviruses. Interestingly, neither cells infected with HCV nor with two different picornaviruses (encephalomyocarditis virus (EMCV) or swine vesicular disease virus (SVDV)) modified the integrity of the protein during the infection, as revealed by Western blot [76]. Instead, Gemin5 was proteolysed in FMDV-infected cells, giving rise to at least two cleavage products, p85 and p57 (Figure 4A). These data suggested that the protease involved in this event was not the 3C protease, which is encoded in the genome of all picornaviruses [77]. In agreement with this, it has been found that the L protease (L^pr^°) of FMDV recognises two sequences, RKAR and TKRL, within Gemin5 (Figure 4A). These sequences contain basic-rich residues, as shown for other L^pr^° substrates [78]. Interestingly, recognition of these sequences is in agreement with the proteolytic products observed during infection [76]. The putative cleavage product (p21) resulting from the TKRL recognition motif has not been detected in infected cells, likely because the cleavage product is not stable (Figure 4A). Further studies indicated that coexpression of L^pr^° and the peptide G5/1078-1438-FLAG containing the RAGHR sequence in transfected cells yielded a p25 proteolytic product. The lack of this cleavage product in double and triple mutants of this motif generated by substitution of basic residues to glutamic acid (RAGHR to RAGEE and EAGEE) demonstrated that L^pr^° is the protease recognising this motif.

The p85 product results from the cleavage at the RKAR sequence [76] and includes both RBS1 and RBS2. Cleavage of p85 at the TKRL sequence yields p57, a fragment that includes RBS1, but not RBS2. Both p85 and p57 interact with the IRES element in UV-crosslink assays (Figure 4B) [30]. A peptide encompassing amino acids 1297–1412, containing only the RBS1 region, has been shown to interact with domain 5, as well.

Overexpression of the p85 product produces a moderate upregulatory effect of IRES-dependent translation [30]. A similar result has been shown with the C-ter polypeptide, containing RBS1 and RBS2 regions (Figure 4B). In contrast, overexpression of the p57 product, which contains RBS1, but not RBS2, has no effect on the efficiency of the IRES-dependent translation; nor does overexpression of RBS1 polypeptide affect the efficiency of IRES-driven translation. This result suggests that RBS2 is crucial to downregulate IRES-dependent translation. In fact, overexpression of the RBS2 polypeptide downregulates IRES-driven translation *in vitro* and in living cells, confirming that RBS2 is the region responsible for the downregulatory effect of the protein [30].

To explain the role of Gemin5 in IRES-dependent translation in infected cells, we propose the following hypothesis (Figure 4B). The full-length form of Gemin5 interacts with the IRES element through its bipartite RBS (RBS1 and RBS2), conferring a maximum binding capacity and downregulating IRES-dependent translation. Proteolysis of Gemin5 in FMDV-infected cells by L^pr^° renders the p85 fragment. This polypeptide, which does not contain the amino-terminal region of the protein, could undergo a local conformational change within the RBS1 domain, resulting in a different crosstalk between the RBS1 and RBS2 moieties. We propose that the local structural rearrangement of the RBSs present in C-ter could be responsible for a moderate upregulation effect of IRES-dependent translation initiation, as observed in p85.

The structural rearrangement of the RBS1 may take place in two different manners. One, the lack of the N-terminal region, may originate a local refolding of the RBS1 domain due its intrinsically unstructured flexibility [30]. Two, following proteolysis, the RBS1 domain may interact with an unknown factor “Y” (protein, RNA or cofactor). The factor “Y” may induce a local conformational change within the RBS1 domain. As the infection advances, proteolysis of p85 gives rise to p57 and an unstable fragment of about 21 kDa (Figure 4A). The p57 fragment is stable in infected cells and does not affect IRES-dependent translation in transfected cells, possibly due to the lack of RBS2. In contrast, the C-terminal fragment p21, which is a negative regulator of IRES activity [30], was undetected in infected cells. In summary, the protein Gemin5 is processed into various polypeptides in infected cells with the characteristic that only stimulators or innocuous fragments for IRES-dependent translation are stable.

## 6. Conclusions

In this review, we have discussed recent advances in the understanding of the functions of Gemin5. This protein performs a key role, not only in the biogenesis of snRNPs, but also in alternative splicing, stress response and translation control. The distinct roles of Gemin5 are related to its capacity to recognise different RNA targets, the snRNAs (by means of the snRNP code), the cap residue (m7 GpppN) of mRNAs or a specific domain within the FMDV IRES element (a short hairpin surrounded by C-, U- or A-rich sequences). According to the identification of the domain of the protein involved in the recognition of the different RNA motifs, the N-terminal region participates in snRNAs recognition, while the most C-terminal region determines not only the interaction with the FMDV IRES, but also harbours the capacity to downregulate translation. Interestingly, the region of the protein containing the repressor domain is not stable in infected cells, indicating that viruses have developed mechanisms to subvert host factors and benefit the viral replication cycle.

Most RNA-binding proteins bear conserved motifs, known as the RBDs. However, neither the N-terminal region nor the C-terminal region of Gemin5 harbours a canonical RBD. The fifth WD repeat domain has been demonstrated to be involved in the recognition of the snRNP code, as well as in the recognition of the cap. In contrast, a bipartite non-canonical RNA binding domain within the C-terminal region is responsible for the interaction with the IRES element and, moreover, for the negative effect on internal initiation. This non-canonical motif, however, appears to have an intrinsically disorganised structure, which can confer flexibility to the protein and, thus, the possibility to select different conformations depending on the function exerted by the protein. To date, the network of RNAs and proteins interacting with this region of Gemin5 is unknown. Future studies aimed at disclosing these networks will undoubtedly reveal new unanticipated roles of Gemin5 in other cellular processes.
